# Xeno‐free self‐assembling peptide scaffolds for building 3D organotypic skin cultures

**DOI:** 10.1096/fba.2022-00026

**Published:** 2022-06-24

**Authors:** Yihua Loo, Andrew C. A. Wan, Charlotte A. E. Hauser, E. Birgitte Lane, Paula Benny

**Affiliations:** ^1^ Institute of Bioengineering and Nanotechnology Singapore; ^2^ King Abdullah University of Science and Technology Division of Biological & Environmental Science & Engineering Thuwal Kingdom of Saudi Arabia; ^3^ Institute of Medical Biology Singapore; ^4^ Skin Research Institute of Singapore Singapore

**Keywords:** bioprinting, in vitro skin models, organotypic cultures, self‐assembling peptides, xeno‐free synthetic bioinks

## Abstract

Organotypic skin cultures represent in vitro models of skin which can be used for disease modeling, tissue engineering, and screening applications. Non‐human collagen is currently the gold standard material used for the construction of the supporting matrix, however, its clinical applications are limited due to its xenogeneic origin. We have developed a novel peptide hydrogel‐based skin construct that shows a pluristratified epidermis, basement membrane, and dermal compartment after 3 weeks of in vitro culture. Peptide‐based constructs were compared to collagen‐based constructs and stratification marker expression was histologically higher in peptide constructs than in collagen constructs. Transepithelial electrical resistance also showed mature barrier function in peptide constructs. This study presents a novel application of the self‐assembling peptide hydrogel in a defined xeno‐free in vitro system.

**Abbreviations**
2D: two dimensional
3D: three dimensional
ADMET: absorption, distribution, metabolism, excretion, toxicity
ANOVA: analysis of variance
ECM: extracellular matrix
FMCG: fast‐moving consumer goods
H&E: hematoxylin and eosin
HDF: human dermal fibroblasts
TEER: trans‐epithelial electrical resistance

## 1 INTRODUCTION

The skin is the largest organ in the human body and functions as a protective barrier to maintain homeostasis and retain water and nutrients while restricting the entry of pathogens.^1^, ^2^ The skin comprises a dermal layer of collagens and glycoproteins primarily containing fibroblast cells plus vascular, lymphoid, and neural structures, an epidermal layer comprised of mostly basal and differentiating keratinocytes and a basement membrane that tethers the two layers together.^3^ Several in vitro models have been developed to mimic the skin for disease modeling, toxicity screens, and skin replacement cell therapy.^4^

Two‐dimensional (2D) cell culture models typically consist of fibroblasts and keratinocytes. However, 3D organotypic skin co‐cultures are a more accurate representation of the skin in vivo. Three‐dimensional (3D) multi‐cellular, tissue mimetic models are powerful experimental platforms that enable the in vitro study of mammalian tissue development, the modeling of human disease, and the evaluation of novel therapeutics.^5^ Organotypic 3D cultures mimic more closely the spatial topography and chemical gradients which results in a better model of in vivo physiology and cellular function including immune system activation, defense response, cell adhesion, and tissue development.^4^ Organotypic skin constructs, in particular, have gained significant research and commercial interest in the face of bans on animal‐tested cosmetic products.^6^ Human cadaveric skin and excised skin from surgical waste are viewed as the gold standard for topical and transdermal permeation models,^7^ although these show high sample‐to‐sample variability.^8^ Animal skin, though easily procured, is inherently different in physiology and gene expression from human skin.^9^ Recent advances in bioprinting have stirred the imagination of tissue engineers. In theory, bioprinting offers a means of preparing customized and complex tissue models, as well as a reproducible manufacturing process to scale up the production of multi‐domain biological constructs that mimic healthy and disease tissue for high‐throughput testing.^10^ This has resulted in significant commercial interest from pharmaceutical and FMCG (fast‐moving consumer goods) companies to bioprint in vitro 3D organotypic skin models for evaluating toxicology, pre‐screening therapeutics, and studying ADMET (absorption, distribution, metabolism, excretion, toxicity). The production of customized disease models that recapitulate phenotype in human patients is also an industrial priority as a major challenge in developing novel therapeutics for skin‐related conditions is the lack of suitable in vitro models for pre‐screening before further evaluation in relevant animal models. Organotypic can mimic specific disease characteristics in a controlled and reproducible manner. Large organotypic skin constructs can also potentially be applied as grafts, fulfilling the unmet clinical needs of burn patients. In summary, organotypic skin constructs have important potential applications as in vitro models of epithelial development and disease, for evaluation of novel therapeutics for skin disease, and as cell‐based grafts for skin injuries such as burns, and chronic wounds.

Despite current scientific advances, there remain some unmet requirements for each of the above applications which include (i) ease of customization, to incorporate different cell types and extracellular matrix (ECM) components to create pared‐down or complex dynamic models; (ii) ability to scale‐up for high‐throughput screening applications, (iii) scale‐up and long‐term storage ability for on‐demand needs. New strategies being evaluated to address these issues include bioprinting (for customization and scaling up production) and molding for preparations of large areas of simple organotypic skin.

A classic approach to the generation of a skin‐mimetic construct in vitro is to first form a base of fibroblasts extracted from the dermis and embedded in a 3D gel matrix of bovine collagen, and then overlay the collagen gel with keratinocytes isolated from the epidermis. Several different biomaterials have been tested as dermal matrices, based on their ability to mimic skin extracellular matrix (ECM). Collagen is currently the most widely‐used biomaterial for making such organotypic full‐thickness skin equivalents, as it contains natural ligands to mediate keratinocyte and fibroblast attachment and proliferation. However, concern about potential immunogenicity and xenopathogenicity of a non‐human component will always restrict the clinical use of bovine collagen‐based organotypic cultures (e.g., for skin grafting), while batch‐to‐batch variations in the collagen reduce the reproducibility of the end product skin equivalent and make quality control, difficult. There is also a major challenge in standardizing collagen hydrogels for organotypic cultures in that the gels are variably remodeled by dermal fibroblasts, which leads to uneven shrinkage of the tissue construct after 3 weeks of culture.

These technical problems drove us to consider the use of self‐assembling synthetic peptide hydrogels as a substitute for non‐human collagen to generate the dermal matrix. Short peptides are versatile building blocks for fabricating supramolecular structures. A specific motif enables ultrasmall peptides with 3–6 amino acids to self‐assemble into helical supramolecular fibers.^11^ This amphiphilic peptide motif consists of a tail of aliphatic nonpolar amino acids with decreasing hydrophobicity and a hydrophilic head group. The peptides undergo a structural transition pathway from random coils to α‐helical intermediates to β‐turn structures with increasing concentration. The β‐turn fibers further condense into meshed 3D nanofibrous networks, which closely resemble the ECM. Due to their strong amphiphilic nature, the peptide networks entrap water, forming 3D nanofibrous hydrogels. Such peptides are highly suitable for tissue engineering applications, due to their outstanding biocompatibility, mild gelation conditions, and excellent mechanical properties. Moreover, they have been investigated as bioink candidates for bioprinting of 3D tissue constructs.^12^ In this study, we use a hexamer peptide, Ac‐ILVAGK‐NH_2_, which demonstrates salt‐enhanced gelation.

The objective of this pilot study was to construct a novel 3D organotypic skin construct using a xeno‐free self‐assembling peptide and incorporating fibroblasts and keratinocytes into the microstructures. We evaluated this novel construct in comparison to the current gold standard in the field. We quantified the trans‐epithelial electrical resistance (TEER) developed in the construct, using an optimized protocol we established to better deal with the challenge of in vitro barrier function development.

## 2 METHODS

### 2.1 Cell culture

Human primary keratinocytes and fibroblasts were isolated from normal human adult female skin obtained from surgical waste skin remnants (abdominoplasty), with written, informed patient consent and full local Institutional Review Board ethics approval. The use of the primary cells was also approved by the Skin Cell Bank IMB‐IRB, Singapore. Fibroblasts were cultured in high glucose DMEM, supplemented with 10% fetal bovine serum and 1% penicillin‐streptomycin. Keratinocytes were expanded in serum‐free keratinocyte media (CnT‐57; Cell‐N‐Tec). All cell culture reagents were purchased from Life Technologies (Carlsbad, CA).

### 2.2 Preparation of collagen‐based constructs

**FIGURE 1:**
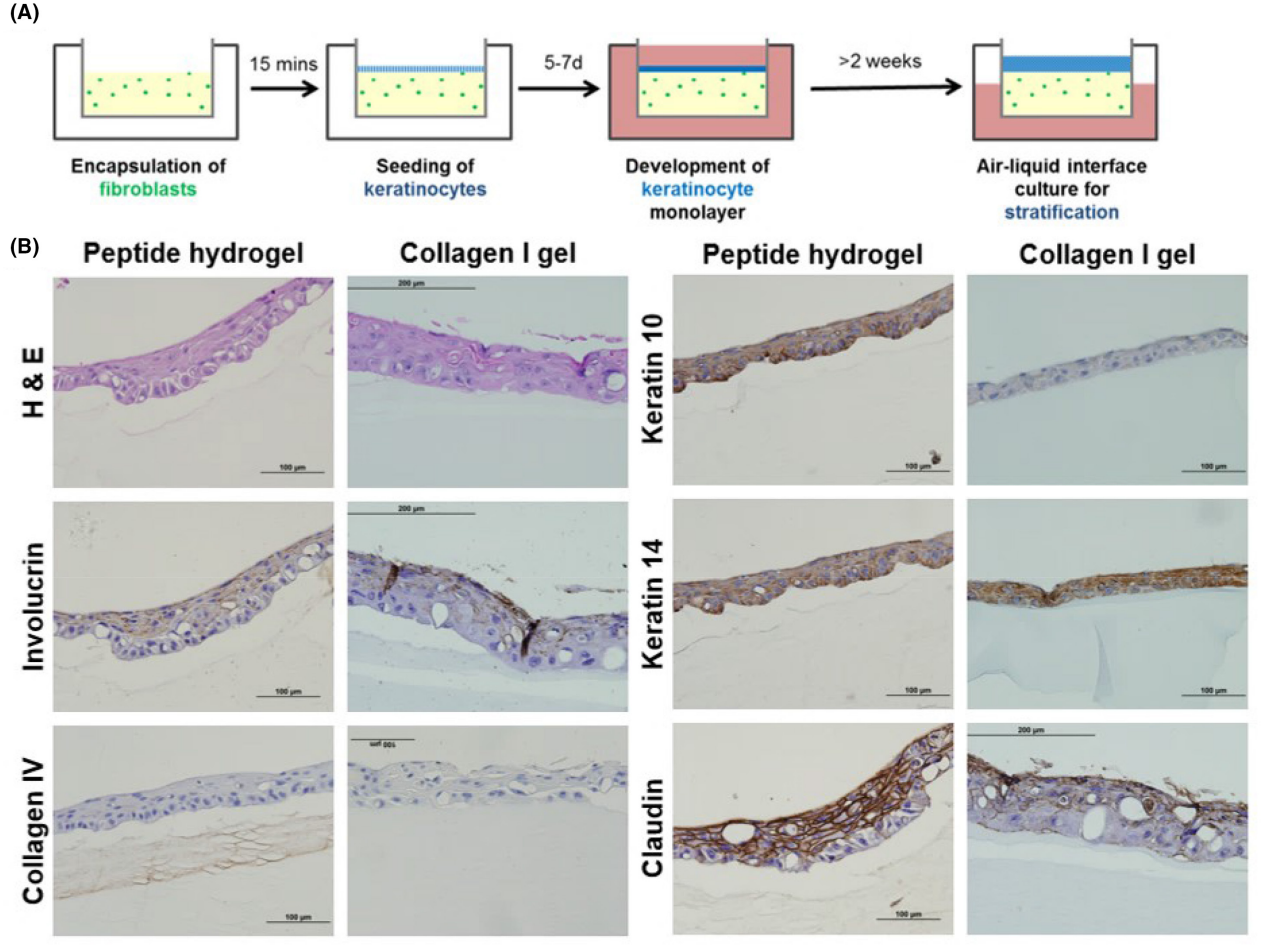
(A) Organotypic culture on peptide‐based vs. collagen‐based matrix. Human dermal primary fibroblasts are encapsulated within the hydrogel (peptide or collagen). After approximately 15 min of gelation, human primary keratinocytes are seeded on top of the hydrogel. After 7 days of submerged culture, confluent keratinocyte monolayers are formed. The construct is raised to the air‐liquid interface, cultured for 2 weeks more, and then harvested. (B) Histology of peptide and collagen constructs. After 14 days of air‐liquid interface culture, organotypic skin cultures were stained for hematoxylin and eosin to determine the construct maturity. A panel of skin biomarkers was also performed. Keratin 14 (basal keratinocyte layer); keratin 10 (suprabasal layers); collagen IV (basement membrane); involucrin (stratification); claudin (tight junctions).

Three milliliters of cold rat tail collagen type I solution (suspended in acetic acid; BD Biosciences) were mixed with 0.5 ml DMEM followed by 0.5 ml 1 M NaOH for neutralization. 1 ml of DMEM containing 10^6^ human dermal fibroblasts (HDF) was added next. 0.5 ml of the collagen–HDF suspension was aliquoted into 12‐mm transwells in a 12‐well plate (Corning) and then transferred to 37°C to allow for gelation for approximately 15 min. After the collagen gel had solidified, human primary keratinocytes were seeded on top of the collagen gel (Figure 1). The constructs were cultured submerged in a medium for 7 days, after which cell culture inserts were raised to the air–liquid interface to promote keratinocyte stratification and differentiation. After 2 weeks, the mature organotypic cultures were harvested for analysis.

### 2.3 Preparation of peptide hydrogel‐based constructs

All peptides used in this study were manually synthesized by the American Peptide Company using solid‐phase peptide synthesis and purified to >95% via HPLC. Peptide solution was first prepared by dissolving lyophilized peptide Ac‐ILVAGK‐NH_2_ in cold sterile water to a stock concentration of 10 mg/ml. To mimic the dermal compartment, 1 ml of sterile water was first mixed with 0.5 ml of 10X PBS and 0.5 ml of cell suspension (containing 10^6^ HDF/ml). This diluted cell suspension was then quickly mixed with 3 ml of cold peptide solution. 0.5 ml of hydrogel was aliquoted into 12‐mm transwells in a 12‐well plate (Corning) and incubated at 37°C for at least 15 min. Following complete gelation, human primary keratinocytes were seeded on top of the hydrogel (Figure 1). The peptide‐based constructs were then cultured and harvested as for the collagen‐based constructs above.

### 2.4 Histological analysis

Samples were fixed in 4% paraformaldehyde. Paraffin embedding was carried out and 4 μm sections were stained with hematoxylin and eosin (H&E) for routine histological evaluation under an Olympus BX51 microscope. Densitometric quantification was performed using ImageJ software.

### 2.5 Trans‐epithelial electrical resistance measurements

Each transwell containing a skin construct was placed within the chamber of an EVOM2 epithelial voltohmmeter (World Precision Instruments). An O‐ring was placed on top of the construct to ensure a standardized surface area of measure and a tight seal for resistance measurements. Media was added to both the inside and outside of the Transwell before resistance was measured. TEER measurements were obtained from cultures at 7 days, 14 days, and 21 days to determine the establishment of skin barrier function in vitro. Before each time point was measured, a blank control was first taken to determine the baseline voltage drift.

## 3 RESULTS

### 3.1 Histological examination of organotypic skin constructs

Organotypic skin constructs were generated using an optimized protocol involving a submerged and air‐liquid interface component, as shown in Figure 1. The submerged component first involved seeding the fibroblasts within the gel and subsequently seeding the keratinocytes on top of the gel. After the fibroblasts and keratinocytes grew to confluence, cultures were lifted to the air‐liquid interface to facilitate epidermal stratification. Constructs were fixed and analyzed through hematoxylin and eosin staining and antibody staining of key basal and suprabasal epidermal components (Figure 1B). While hematoxylin and eosin staining showed mature basal and suprabasal epidermal layers, the peptide hydrogel showed a tighter basal epidermal layer and more mature epidermal stratification. Visualization of fibroblasts was not captured, possibly due to cell loss during the histological processing steps, as the hydrogel base was composed of self‐assembling peptides which easily dissolve in ethanol. Involucrin and keratin 10, markers of epidermal stratification, are more prominent in peptide hydrogels as compared to collagen hydrogels. Collagen IV, a basement membrane marker, shows positive staining in peptide hydrogels, suggesting that the fibroblasts and keratinocytes culture in a peptide construct were successfully able to secrete basement membrane components. Collagen IV deposition was observed to localize in the fibroblast zone closest to the epithelial layer. Claudin, a marker of tight junctions, is more significantly present in peptide hydrogels as compared to collagen hydrogels. Keratin 14 staining, a marker of the basal epidermal layer, is prominent in both hydrogel constructs. Densitometric quantification of the histological micrographs is summarized in Figure S1.

### 3.2 Quantitative evaluation of barrier function

**FIGURE 2:**
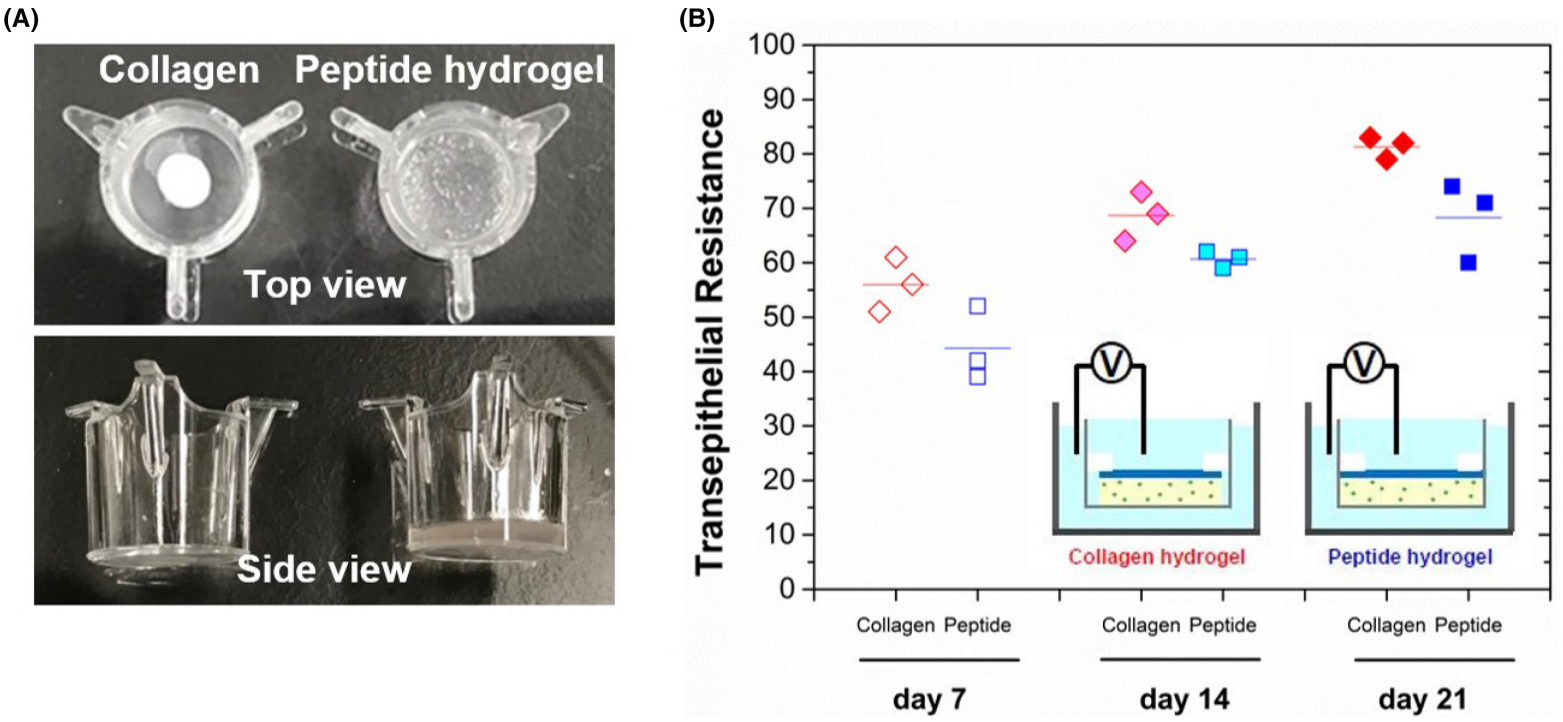
(A) Macroscopic appearance of collagen vs peptide hydrogel constructs. Seen in the culture inserts, top view observation of the collagen hydrogel shows extensive shrinkage after 3 weeks of tissue culture. In contrast, peptide hydrogels did not shrink. Side view images show substantial thinning of collagen constructs which were not seen in peptide constructs. (B) TEER measurements of organotypic skin constructs developed using collagen or peptide hydrogels. TEER measurements were obtained at 3 time points (7 days, 14 days, and 21 days) to track the skin barrier formation in vitro. TEER rises with time on culture as the skin barrier develops. At all 3 time points, collagen constructs showed slightly higher TEER readings than the peptide constructs. Red data points = collagen constructs, blue data points = peptide constructs.

Images of the hydrogels within the transwells were captured upon completion of the culture protocol and before fixation, as shown in Figure 2. Top views of the peptide hydrogel show an absence of gel shrinkage, while that of the collagen hydrogel shows marked shrinkage. Side view comparisons show a thick peptide hydrogel even after several weeks, however, the collagen hydrogel was observed to be very thin.

Trans‐epithelial electrical resistance *(*TEER) analysis of the constructs shows a gradual increase in TEER over the three time points (day 7, 14, 21). Of interest, at all three time points, collagen constructs showed higher TEER readings as compared to peptide hydrogel constructs. ANOVA was performed and a statistical significance of *p* < 0.05 was observed between hydrogels (peptide vs collagen; *p* = 0.0007) and days of harvest (day 7 vs 14 vs. 21; *p* = 0.00001).

## 4 DISCUSSION

Organotypic constructs aim to mimic natural full‐thickness skin tissue by encapsulating human dermal fibroblasts within a biomimetic hydrogel that mimics the dermis and supports the proliferation and maturation of human dermal keratinocytes cultured on its surface. Collagen is currently the most commonly used matrix for building organotypic full‐thickness skin. It is regarded as a good scaffold material since it is the primary component of skin tissue. However, its xenogeneic origin currently limits its use for medical applications.

The peptide hydrogel supports the proliferation of keratinocytes into a confluent monolayer and subsequent maturation of keratinocyte monolayers to a stratified epidermis with distinct basal and suprabasal layers. Upon transition from basal to suprabasal layers, keratinocyte morphology changes from cuboidal to increasingly flattened, ultimately developing into the stratum corneum. Keratin expression shifts from keratins K5 and K14 in basal layer cells to keratins K1 and K10 in suprabasal layers. Upon histological analysis of both the peptide and collagen constructs, hematoxylin, and eosin staining shows the change of keratinocyte nuclear profile from a round to a horizontal ovoid shape between the basal to the suprabasal layer as seen in Figure 1. While keratin 14 is expressed in both peptide and collagen constructs, more keratin 10 was observed in peptide constructs. Also observed in peptide constructs was the expression of collagen IV, which is a critical skin basement membrane protein. Claudin, a marker of tight junctions, was observed to show higher expression in peptide‐based skin constructs, suggesting more significant maturation was taking place in the peptide‐based organotypic constructs than in the collagen‐based constructs within the same in vitro culture timeframe.

There was no shrinkage of the peptide construct, even after 2 weeks of culture at the air‐liquid interface as seen in Figure 2, which indicated that fibroblasts are unable to remodel the peptide matrix. In contrast, collagen constructs shrank significantly after 1 week of submerged culture. After 2 weeks of culture at the air‐liquid interface, collagen gels were approximately a quarter of their original size. The volume of shrinkage was also uneven as the 3D shape was biconcave. The uneven thickness and lack of predictability of the collagen‐based construct surface area would also present challenges for measuring trans‐epithelial transport.

Construct area and thickness are major factors that influence transepithelial transport measurements, such as permeability. In order to compare constructs of varying sizes, we developed customized silicon O‐rings to standardize the area of measurement. They also served to seal the edges of the transwell and enabled us to utilize standard assays for quantifying barrier function. Trans‐epithelial electrical resistance (TEER) was chosen as a surrogate to quantify permeability and hence indirectly evaluate functional epithelial differentiation. This is a non‐destructive measurement technique, which can be used to track the development of a single sample, and also serves as a quality control benchmark to determine the barrier function of a given construct prior to drug testing.

We observed a gradual increase in TEER readings for both peptide and collagen gels over time, and this increase in electrical resistance corresponds to an increase in barrier function (Figure 2B). Collagen construct readings were generally higher than peptide construct readings, even though the constructs were thinner. This could possibly be attributed to a thickened stratum corneum, due to a higher local density of skin cells caused by the collagen hydrogel shrinkage.

The novel use of the peptide hydrogel as a matrix for organotypic skin cultures has several advantages over conventional collagen gels. First, it is xeno‐free, which with the use of a serum‐free tissue culture medium, allows the establishment of a fully defined in vitro culture system for the production of tissue culture‐generated skin constructs for transplantation. This would address unmet clinical needs for cell‐based therapies to manage burn injuries and chronic skin wounds. Secondly, the peptide hydrogel is amenable to molding technologies, and hence is easy to scale up, but is also accessible to research groups without access to bioprinter setups. Thirdly, it could function as a biomimetic with a nanofibrous architecture. While it resembles ECM and collagen, the peptide is not extensively remodeled like collagen. While the peptide hydrogel described here currently lacks the signaling motifs of collagen, this can be easily incorporated by conjugation, and these models could then form the basis of studying cell‐ECM interactions in vitro.

## 5 CONCLUSION

The model presented here is amenable to many future applications. It can be expanded with the incorporation of additional cell types to mimic more complex healthy and diseased skin systems; it can also be used for developing cell‐based therapies for wound healing. It lends itself to miniaturization of organotypic for high‐throughput screening assays, as a bioink for bioprinting, incorporation into microfluidics for real‐time permeability, and drug ADMET assays. It is, therefore, exceptionally flexible in a wide range of advanced tissue engineering applications.

## AUTHOR CONTRIBUTIONS

Y.L. and P.B. conceived the idea of the study, designed, and executed the experiments with input from A.C.A.W., C.A.E.H., and E.B.L. The manuscript was written with contributions from all authors. All authors have given approval to the final version of the manuscript.

## FUNDING INFORMATION

This work was supported by the Institute of Bioengineering and Nanotechnology, Institute of Medical Biology, and Biomedical Research Council (Agency for Science, Technology and Research, Singapore). Yihua Loo was also supported by the A*STAR BMRC YIG Grant 14/1/07/51/011.

## ETHICS APPROVAL AND CONSENT TO PARTICIPATE

Cells were obtained from waste tissue with informed patient consent and full local Institutional Review Board ethics approval. All methods were performed in accordance with the local guidelines and regulations.

## CONSENT FOR PUBLICATION

All authors have given approval to publish the manuscript.

## COMPETING FINANCIAL INTERESTS

The authors declare no competing financial interests.

## Supporting information


Appendix S1
