# Supplementary figures and images for: Xeno‐free self‐assembling peptide scaffolds for building 3D organotypic skin cultures
Source: FASEB Bioadv. 2022 Jun 24;4(10):631–7. doi: 10.1096/fba.2022-00026 (PMC9536085; doi:10.1096/fba.2022-00026)

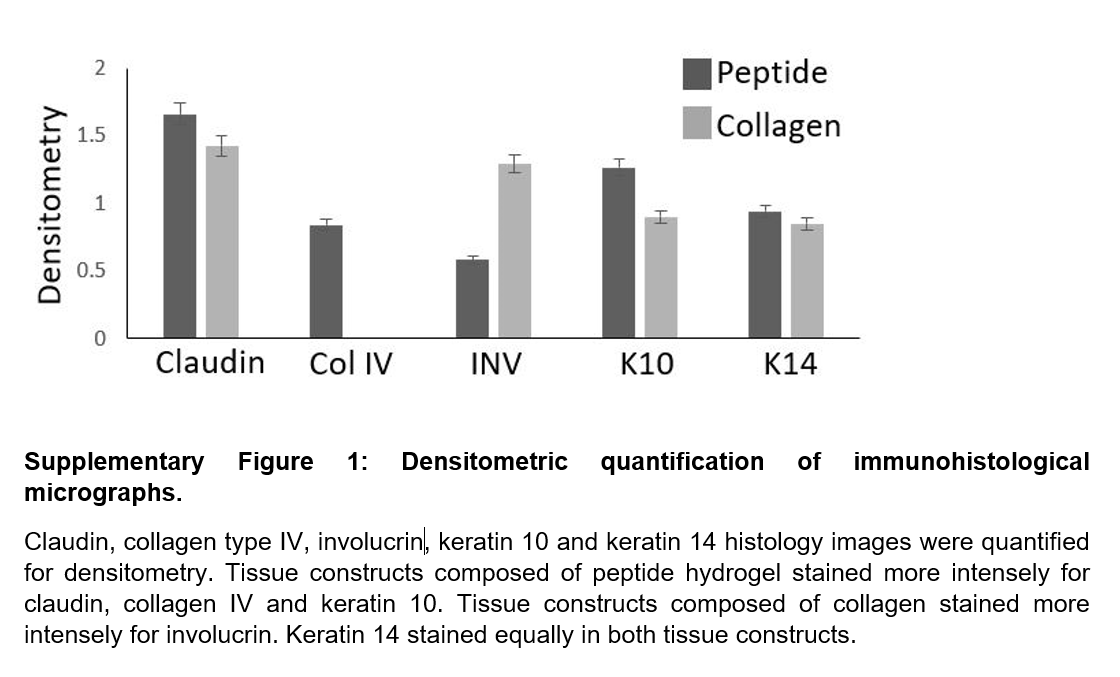

Supplement: Supplementary file 1 — Appendix S1 [file FBA2-4-631-s001.tif]
